# Matching comprehensive health insurance reimbursements to their real costs: the case of antenatal care visits in a region of Peru

**DOI:** 10.1186/s12962-015-0042-z

**Published:** 2015-09-19

**Authors:** Daniel Cobos Muñoz, Kristian Schultz Hansen, Fern Terris-Prestholt, Fiona Cianci, José Enrique Pérez-Lu, Aldo Lama, Patricia J. García

**Affiliations:** Swiss Tropical and Public Health Institute, Basel, Switzerland; University of Basel, Basel, Switzerland; Department of Global Health and Development, London School of Hygiene and Tropical Medicine, London, UK; Department of Public Health, HSE Carlow/Kilkenny, Lacken, Kilkenny, Ireland; School of Public Health and Administration, Universidad Peruana Cayetano Heredia, Lima, Peru; Regional Health Directorate of Callao, Regional Government of Callao, Callao, Peru

**Keywords:** Antenatal care, Cost, Social health insurance, Sustainability

## Abstract

**Background:**

Prepaid contributory systems are increasingly being recognized as key mechanisms in achieving universal health coverage in low and middle-income countries. Peru created the Seguro Integral de Salud (SIS) to increase health service use amongst the poor by removing financial barriers. The SIS transfers funds on a fee-for-service basis to the regional health offices to cover recurrent cost (excluding salaries) of pre-specified packages of interventions. We aim to estimate the full cost of antenatal care (ANC) provision in the Ventanilla District (Callao-Peru) and to compare the actual cost to the reimbursement rates provided by SIS.

**Methods:**

The economic costs of ANC provision in 2011 in 8 of the 15 health centres in Ventanilla District were estimated from a provider perspective and the actual costs of those services covered by the SIS fee of $3.8 for each ANC visit were calculated. A combination of step-down and bottom-up costing methodologies was used. Sensitivity analysis was conducted to test the uncertainty around estimated parameters and model assumptions. Results are reported in 2011 US$,

**Results:**

The total economic cost of ANC provision in all 8 health centres was $569,933 with an average cost per ANC visit of $31.3 (95 % CI $29.7–$33.5). Salaries comprised 74.4 % of the total cost. The average cost of the services covered by the SIS fee was $3.4 (95 % CI $3.0–$3.8) per ANC visit. Sensitivity analysis showed that the probability of the cost of an ANC visit being above the SIS reimbursed fee is 1.4 %.

**Conclusion:**

Our analysis suggests that the fee reimbursed by the SIS will cover the cost that it supposed to cover. However, there are significant threats to medium and longer term sustainability of this system as fee transfers represent a small fraction of the total cost of providing ANC. Increasing ANC coverage requires the other funding sources of the Regional Health Office (DIRESA) to adapt to increasing demand.

**Electronic supplementary material:**

The online version of this article (doi:10.1186/s12962-015-0042-z) contains supplementary material, which is available to authorized users.

## Background

Financing systems that encourage risk-pooling and pre-payment, such as public health insurance, are increasingly recognized as key mechanisms in achieving universal health coverage in low and middle-income countries [1]. They have been shown to improve financial protection and increase access to health services in a variety of settings with different contextual and structural characteristics [2]. The nature of the purchasing arrangements between the prepaid scheme and service providers is an essential factor to their success. An optimal payment system should induce providers to offer high quality services that are responsive to patients’ needs and provided in an efficient way.

In 2001, the Peruvian Government created a comprehensive health insurance package for the poor called the Seguro Integral de Salud (SIS), This is mainly funded from general taxes and aims to expand health service use amongst the poor, by eliminating user fees [3]. The SIS transfers funds on a fee-for-service basis to the DIRESA (Spanish acronym for Regional Health Office) to provide a pre-specified package of interventions, including antenatal care (ANC) services [4]. The fees were estimated in 2003 to cover recurrent cost of services and assuming that personnel cost were covered by the ministry of health. The technical aspects of how they were calculated or if they have been adjusted for inflation are not clear as this information is not open to the public.

In a fee-for-service (FFS) payment mechanism, each service provided to patients is paid for individually. This can create an incentive for health care providers to increase the volume of patients seen and the number of services provided to each patient. This can sometimes become a perverse incentive and lead to either overproduction of services to maximize income (supplier induced demand) or a disproportionate provision of more profitable services [5].

The actual cost of the service provided must be closely related to the fee reimbursed for these financing mechanism to be sustainable. In Peru, few attempts have been made to estimate the actual cost incurred by health providers for the interventions covered by the SIS. Cid et al. found that the actual cost of providing paediatric epilepsy treatment was 7 % less than the fee reimbursed by the SIS and that the cost of treating acute bronchitis was 28 % more than its respective fee [6]. Alvarado et al. calculated the cost of some of the most frequent diagnostics at a paediatric intensive care and compared these to the SIS fees, observing cost up to 32 % higher in some cases [7]. Both these studies only examined the cost of treatment provision and did not include comprehensive overhead costs. To the best of our knowledge, our study is the first to describe the total economic cost of providing a preventive antenatal care (ANC) service in Ventanilla, a peri-urban district in Peru, and compare it to the reimbursement rates provided by SIS in 2011. We hope to strengthen the evidence-base to streamline SIS and further the achievements of its goals.

## Methods

### The setting

Ventanilla is the largest of districts of the Callao Region (Lima). It is an urban location with an estimated population of 389,440 [8]. It was chosen as the site of implementation of “Brighter Futures”. This is a project that aims to understand the factors that influence the introduction of point of care diagnostics to improve maternal and child health in there different geographic regions of Peru (coast, Andes and jungle). Ventanilla District was selected as the representative district of the coastal region of Peru.

In 2011 the Ministry of Health (MoH) network in Ventanila District consisted of 4 sub-networks (3 de Febrero, Angamos, Luis Felipe and Marquez) with one district hospital and 15 health centres in total. We purposively sampled two health centres from each sub-network to capture the expected maximum variation in costs based on a number of indicators, such as, complexity of services provided, catchment size, opening hours and number of ANC visits performed (see Additional file 1: Table S1 for more information on their characteristics).

### The intervention: antenatal care (ANC)

ANC is a combination of different preventive interventions provided to pregnant women before delivery and “…includes education, counselling, screening and treatment…” [9]. The National Reproductive Health Guidelines suggests a minimum of six antenatal care visits during pregnancy to complete the essential package of interventions [10] which, in Peru, is provided through primary health care centres.

The package offered in Ventanilla District does not vary greatly among its health centres. At the initial ANC visit, all attendees must register in the SIS to avail of these services free of charge. Enrolment entails visits to admission services, the SIS office, the pharmacy and a self-funded echography to prove pregnancy, unless the pregnancy is obvious. Once enrolled women are first triaged and then, seen by a midwife in the ANC office. The midwife performs a clinical exam, a rapid syphilis and HIV test and a tetanus vaccination as required. This is followed by a nutrition, a dental care and a psychological assessment. All subsequent visits only involve clinical assessment by the midwife in the ANC office, with prescription of iron and folic acid as required. At the fourth or fifth visit, women also undergo formal HIV and syphilis testing in the laboratory and an ultrasound scan. The visit schedule and interventions provided by various departments are shown and in Table 1.

**Table 1:** Interventions that must be provided to healthy women during pregnancy

Intervention	Visit	Service involved
Midwife ANC attendance^a^	All visits	ANC office, triage, admission and pharmacy
Laboratory tests^b^	1st and 5th visits	Laboratory
Tetanus vaccination	As soon as possible	Immunization service
Iron plus folic acid	All visits from 4th month of gestational age	ANC office and pharmacy
Nutrition screening	1st	Nutrition service
Psychology screening	1st	Psychiatric service
Dental care screening	1st	Dental care service
Echography	1st and 5th visits	Echography service

^a^Clinic history, vital signs, risk factor assessment for complicated pregnancy, iron administration and syphilis and HIV rapid test (this only in the 1st visit)

^b^1st visit: Haemoglobin or haematocrit, Rh factor, glucose and urine test; 5th visit: equal to 1st visit plus RPR and HIV test

The SIS reimburses the DIRESA on a fee-for–service basis (i.e. per ANC visit) to cover some of the cost of this service [11, 12]. Payment is done retrospectively based on the regional reports of the numbers of services provided. The reimbursement fee for each ANC visit was $3.80 in 2011 [13]. This is supposed to cover all recurrent costs of enrolment, triage and the visit to the ANC office, including rapid tests and iron supplements but excluding all salaries and capital costs [14]. Our analysis focuses on this reimbursement fee.

Of note, there is a separate reimbursement fee schedule for other components of ANC provision, such as, $9.55 for each laboratory test performed during pregnancy and $5.73 for each obstetric echography [13]. The DIRESAs also receive funds from two other sources: direct government transfers and fees paid by uninsured patients at the facility level. Most of the government funds are earmarked to cover personnel cost (“Nombrados” and “Contratados” type of contract) and pharmaceuticals, while facility level user fees can be used more flexibly [15].

### Data collection and cost analysis

A field visit was conducted between July and August 2012 to retrospectively collect output and cost data from each of the eight health centres selected. Activity data for each clinic was collected from its health information system and included number of pregnant women seen and services provided. Costs incurred during 2011 calendar year were collected from a provider’s perspective and classified into capital and recurrent costs. Cost data were collected in 2011 Peruvian New Soles (PEN) and reported in 2011 US$ using an exchange rate of 0.382 PEN to 1.00 US$ [16].

Capital costs (building, furniture and equipment) were collected during individual health facility visits. Building costs were estimated by applying the construction cost per square meter to the size of the health facility as detailed in the actual plan of the building. The most recent equipment inventory available for each health facility was used (December 2010) and each item valued using estimates provided by the DIRESA. Annualised costs were calculated using a 5 % discount rate and expected lifespan for building, furniture and equipment of 30, 10 and 5 years respectively [17].

Most of the recurrent costs, such as, utilities, medications and consumables, were retrieved from the Callao Regional Health Office accounting system for each of the eight health facilities. A list of personnel involved in ANC provision was compiled during the field visit. Their job titles, respective duties and time spent on ANC provision was also collected. Personnel costs, including allowances, were provided by the DIRESA.

The full annual cost of providing ANC services in 2011 and the unit cost per ANC visit in each health centre studied were estimated using a step-down costing methodology combined with a bottom-up strategy. We also estimated the cost per visit of those activities that are covered by the ANC visit SIS fee of $3.8. As described previously, this fee only covers recurrent cost of the admission service, SIS office, triage, CRED (vaccination office) and the ANC office, excluding salaries. The interventions included in each cost estimate can be seen in Table 2.

**Table 2:** Different interventions included in the unit cost estimates

	Full ANC visit	SIS fee covered services	1st ANC visit	ANC visit other than the 1st
Attendance to ANC office^a^	X	X^e^	X	X
General support services^b^	X	X^e^	X	X
1st Laboratory test^c^	X		X	
2nd Laboratory test^d^	X			X
Echography	X		X	
Psychology screening	X		X	
Dental care screening	X		X	
Nutrition screening	X		X	

^a^Syphilis and HIV rapid test, iron and folic acid administration and midwife attendance

^b^Admission, Triage, SIS office (only first ANC visit) and tetanus vaccination

^c^Haemoglobin, haematocrit, Rh factor, glucose and urine test

^d^Equal to first laboratory test plus RPR and HIV test

^e^Only recurrent cost and excluding salaries

Step-down costing methodology was used to allocate joint costs of the health centre to the different activities in a step-wise fashion [18, 19]. Services and departments of the health centre were categorised into three cost centres according to their activities and following the organisational structure of the facility: overhead (administration, accounting, health information unit, logistics and security), support (admission, laboratory, echography, pharmacy and SIS office), and final services (triage, ANC office, vaccination service…). Allocation criteria were developed based on the data collected during the field visit, to reflect actual resource utilisation. For instance, relevant staff were asked to estimate the amount of time they spent on each activity. These estimates were used to allocate corresponding staff costs (salaries) to each activity.

More information on the sources of cost data, allocation criteria and the cost categories collected can be found in Additional file 1: Tables S2, S3. All data were compiled, stored and analysed using Microsoft Excel 2013.

As a first step, the total capital and recurrent cost were distributed across each cost centre using specific allocation criteria described above. In the second step, overhead costs were allocated to support and final services (as well as to overhead activities in a simultaneous fashion) [19]. Next, the costs of support services were allocated to the final services and, lastly, a proportion of final services costs (vaccination, triage, nutrition service, psychology service, dental care and health promotion) were allocated specifically to the ANC provision cost.

In addition, a bottom-up methodology was used to estimate the cost of some of the resources used at individual patient level. The unit costs of rapid HIV and syphilis testing and, of iron and folic acid supplementation, were applied to the number of pregnant women that received them. The fees reimbursed by the SIS for each laboratory test and the cost estimate of echography in Peru Korea health centre, were used as respective unit cost estimates. These were then applied to the number of tests and ultrasounds performed in 2011 to calculate the total and per visit cost of full ANC provision in each facility. In order to calculate the cost per ANC visit in the Ventanilla District as a whole, we used the total costs of ANC provision at all eight facilities sampled and divided it by the total number of women seen at those facilities in 2011.

All patient details were anonymized and de-identified prior to analysis. The Ethics Committee at the London School of Hygiene and Tropical Medicine approved the study.

### Sensitivity analysis

An univariate sensitivity analysis was performed to account for the uncertainty around some of the input estimates used. Table 3 shows the parameters included in the sensitivity analysis and the range of values used. In addition, a probabilistic sensitivity analysis was conducted where parameters were simultaneously sampled from a uniform distribution between the maximum and the minimum possible value. The mean ANC unit cost and 95 % CI were estimated using 1000 iterations of the model.

**Table 3:** Parameters included in the sensitivity analysis

Parameter	Base case	Range (Lo–Hi)	Source/notes
Discount rate	0.05	0.03–0.1	Base case as defined in [17]. Range defined by the author
Coverage of laboratory test	70 %	50–100 %	Assumption based on the information provided by the staff of the health centres Coverage of laboratory test among women attending to the ANC office
Coverage of administration of iron plus folic acid	70 %	50–100 %	Assumption based on the information provided by the staff of the health centres % of pregnant women attending to the ANC office that received iron plus folic acid
Coverage of echography	Specific for each facility	±20 %	Estimated for each health centre using WawaRed records with data from the digital clinic history used for maternity services Estimate for each facility in Table 4
Price of rapid syphilis test	$1.80	±20 %	Provided by the DIRESA. Range defined by the author
Price of rapid HIV test	$2.15	±20 %	Provided by the DIRESA. Range defined by the author
Price of iron plus folic acid	$0.83	±20 %	Provided by the DIRESA. Range defined by the author
Cost of electricity, telephone, office consumables and cleaning	Specific for each facility	±20 %	Estimated by the DIRESA for each health centre
Recurrent cost of laboratory tests for pregnant women (excluding salaries)	$9.55	±20 %	Assumed to be equal to the fee reimbursed by the SIS for this service
Cost of Echography	$18.40	±20 %	Estimated with data from Peru-Korea health centre. Range defined by the author
Time of consultation per ANC visit in the ANC office	16.2 min	15.4–17.0 min	As estimated by Pérez-Lu et al. plus the time used for the PAP smear screening
% of time in the other final services allocated to ANC provision	Specific for each facility	±20 %	Estimated by the staff working in each service in each facility (reported value can be seen in in Additional file 1: Table S4)

## Results

Among the approximately 8737 pregnant women in Ventanilla District in 2011, ANC was provided to 6796 women. Of these, 2,802 women attended one of the eight health centres sampled resulting in a total of 18,220 visits performed in 2011. Each woman received an average of 6.5 ANC visits. The coverage of the different services provided to pregnant women attending to ANC can be seen in Table 4.

**Table 4:** Coverage of interventions among pregnant woman attending to ANC in 2011

Health centre	Number of women attended	Coverage of different interventions (%)					
		Syphilis rapid test	HIV rapid test	Dental care	Psychology screening	Tetanus vaccination^a^	Echography^b^
3 de Febrero	423	100.0	100.0	66.9	100.0	42.1	73.3
Peru Korea	525	100.0	96.2	29.0	99.6	49.7	76.8
Defensores	231	100.0	100.0	24.7	100.0	67.5	54.9
Mi Peru	615	100.0	100.0	64.1	98.4	69.4	83.1
Santa Rosa	308	100.0	100.0	69.5	100.0	60.1	92.7
Villa los Reyes	304	100.0	100.0	57.9	100.0	62.2	80.6
Marquez	329	100.0	100.0	111.9	96.4	45.3	82.3
Ventanilla Baja	67	100.0	100.0	44.8	100.0	41.8	84.0
Total sampled facilities	2802	100.0	99.3	59.7	99.1	56.1	–

Source: data provided by the Health Information System in Ventanilla District

^a^Women completely vaccinated previously did not need this intervention

^b^Estimated using WawaRed records with data from the digital clinic history used for maternity services

The total economic cost of ANC provision in the 8 sampled facilities was 569,933. It varied widely between the study facilities ranging from $23,231 in Ventanilla Baja to $110,000 in Peru Korea (Table 5). The major share of the costs were salaries and medical supplies (drugs and diagnostics), representing on average 77.8 and 13.5 % of the total cost respectively. Capital cost ranged from $2960 in Ventanilla Baja to $10,967 in Peru Korea representing on average 10.4 % of the total cost.

**Table 5:** Total Cost of the provision of ANC (US$ 2011)

	3 de Febrero	Defensores	Marquez	Mi Peru	Peru Korea	Santa Rosa	Ventanilla Baja	Villa de los Reyes	Total
Recurrent cost	82,393	33,166	89,122	77,778	99,033	61,577	20,271	47,201	510,542
Distributed across (%)									
Salaries	77.3	72.5	84.7	74.2	78.5	76.9	79.6	73.6	77.8
Medicine and pharmaceutical^a^	12.5	11.3	6.7	15.0	11.0	12.0	6.6	13.5	11.3
Building and maintenance^a^	4.6	7.6	2.8	3.9	3.7	4.8	7.5	5.9	4.4
Non drugs consumables^a^	2.1	2.7	1.5	3.1	2.0	2.0	1.5	2.6	2.2
Office consumables^a^	0.8	2.0	1.0	0.9	1.3	2.0	2.8	0.8	1.2
Cleaning materials^a^	0.4	1.3	0.4	0.5	0.5	0.6	1.1	0.7	0.6
Telephone^a^	0.1	0.2	1.1	0.1	0.5	0.2	0.2	0.1	0.4
Electricity/water^a^	2.2	2.4	1.8	2.1	2.6	1.4	0.8	2.7	2.1
Capital cost	8591	4664	7810	8483	10,967	8346	2960	7571	59,391
Distributed across (%)									
Buildings	70.9	87.0	50.7	58.3	53.4	57.4	82.5	59.0	61.6
Equipment	24.8	10.5	44.1	36.4	44.2	39.7	14.4	38.2	34.7
Furniture	4.3	2.5	5.2	5.3	2.4	2.9	3.1	2.8	3.6
Total cost	90,984	37,829	96,932	86,261	110,000	69,923	23,231	54,772	569,933

Cost reported in this table represent the total cost of all services provided in the ANC visits (financial year 2011): Attendance to ANC office, general support services, laboratory, echography, psychology, dental care and nutrition service

^a^Covered by the ANC visit SIS reimbursement fee (attendance to ANC office and the general support services). The total cost of these services for all health facilities were $61,146

The allocation of costs using the step-down methodology resulted in the burden of resources consumed being shared roughly equally among final services (dental care, psychiatric services, nutrition, triage and immunization service), support services (SIS office, pharmacy, laboratory and echography services) and the ANC office. They represented on average 36.2, 35.0 and 23.5 % respectively of the total cost of the ANC provision, while overhead cost represented 5.3 %.

Table 6 shows the cost per ANC visit for the different health centres examined. The average cost per ANC visit across the eight facilities is $31.3 with a 95 % CI between $29.7 and $33.5. Some variability can be found among the health centres with the unit cost of ANC provision ranging from $20.6 in Mi Peru to more than double in Ventanilla Baja ($48.4). The 1st ANC visit is more than three times as expensive as the rest of the visits in our study ($75.3 and $21.0 respectively).

**Table 6:** Unit cost of ANC expressed as cost per ANC visit (US$ 2011) and summary statistics

	3 de Febrero	Defensores	Marquez	Mi Peru	Peru Korea	Santa Rosa	Ventanilla Baja	Villa de los Reyes	All health centres
Unit cost									
Full cost of ANC^a^ (CI 95 %)	35.8 (33.6–38.8)	28.1 (26.3–30.5)	45.4 (43.6–47.6)	20.6 (19.4–22.2)	35.7 (33.6–38.4)	30.8 (29.3–32.8)	48.4 (46–51.2)	25.3 (23.7–27.3)	31.3 (29.7–33.5)
Cost excluding salaries and capital cost (CI 95 %)^b^	3.8 (3.3–4.3)	4.1 (3.6–4.6)	3.7 (3.3–4.1)	2.3 (2.0–2.6)	3.7 (3.3–4.2)	3.3 (2.9–3.7)	5.6 (4.9–6.3)	3.0 (2.7–3.4)	3.4 (3.0–3.8)
Statistics									
Number of ANC visits	2543	1344	2136	4194	3084	2270	480	2169	18,220
ANC visits per women attended	6.0	5.8	6.5	6.8	5.9	7.4	7.2	7.1	6.5
Ratio ANC visit per full time midwife per year	963	974	1504	2087	1904	946	369	1356	1268

^a^Cost of all services provided during the ANC visits

^b^Cost of those services covered by the ANC visit SIS reimbursement fee (attendance to ANC office and general support services)

**Fig. 1:**
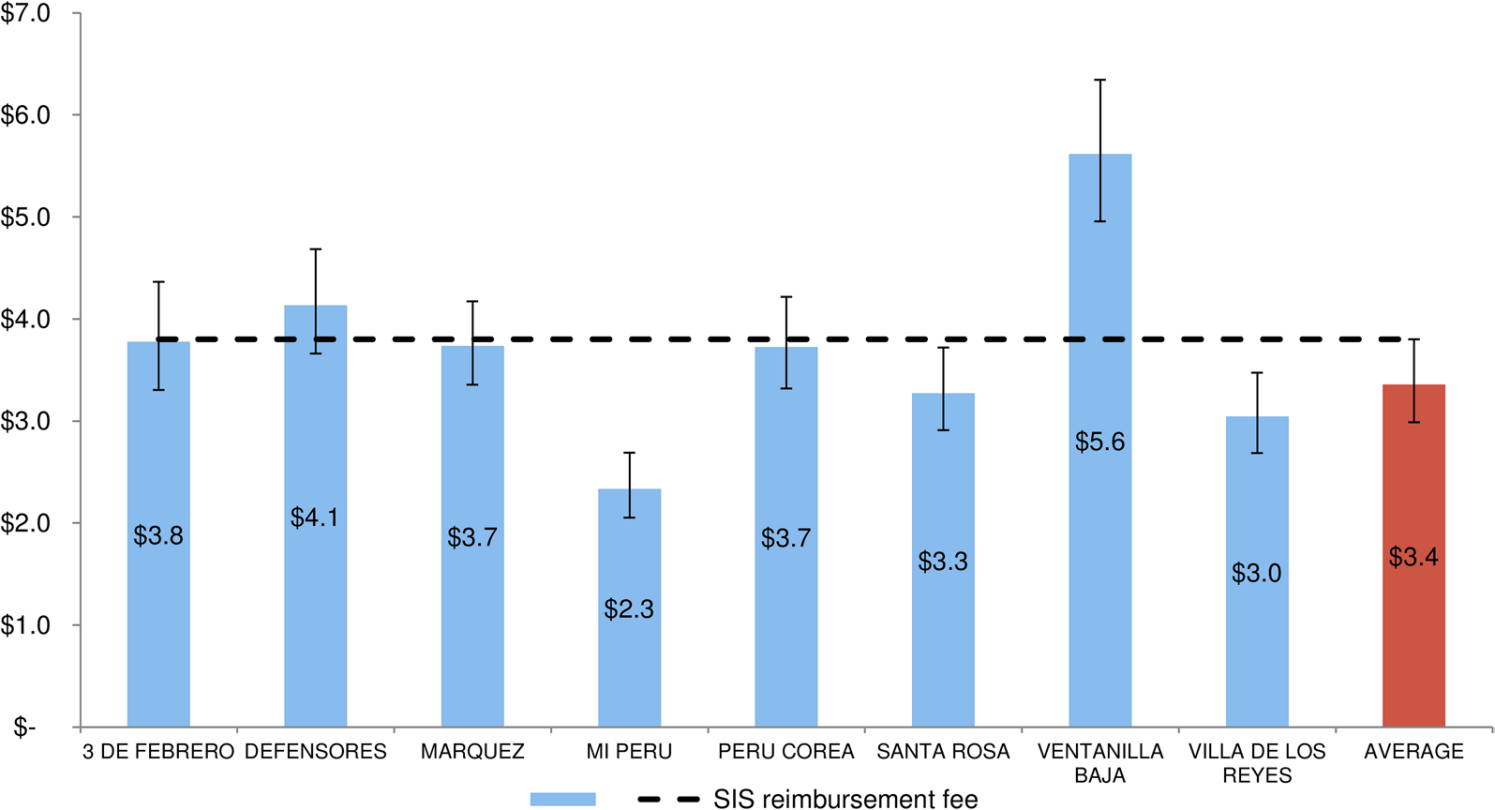
Unit costs with 95 % CI of the services covered by the fee from the SIS

The total actual cost of those services covered by the SIS for each ANC visit (recurrent cost excluding salaries of the attendance to ANC office and general support services) in 2011 was found to be $61,146 that represent 10.7 % of the total economic cost of providing ANC. This translated to an actual cost per ANC visit of $3.4 (95 % CI $3.0–$3.8), ranging from $2.3 in Mi Peru to $5.6 in Ventanilla Baja. As shown in Fig. 1, only 3 of the 8 health centres (3 de Febrero, Defensores and Ventanilla Baja) would have higher cost than the fee reimbursed.

### Sensitivity analysis

**Fig. 2:**
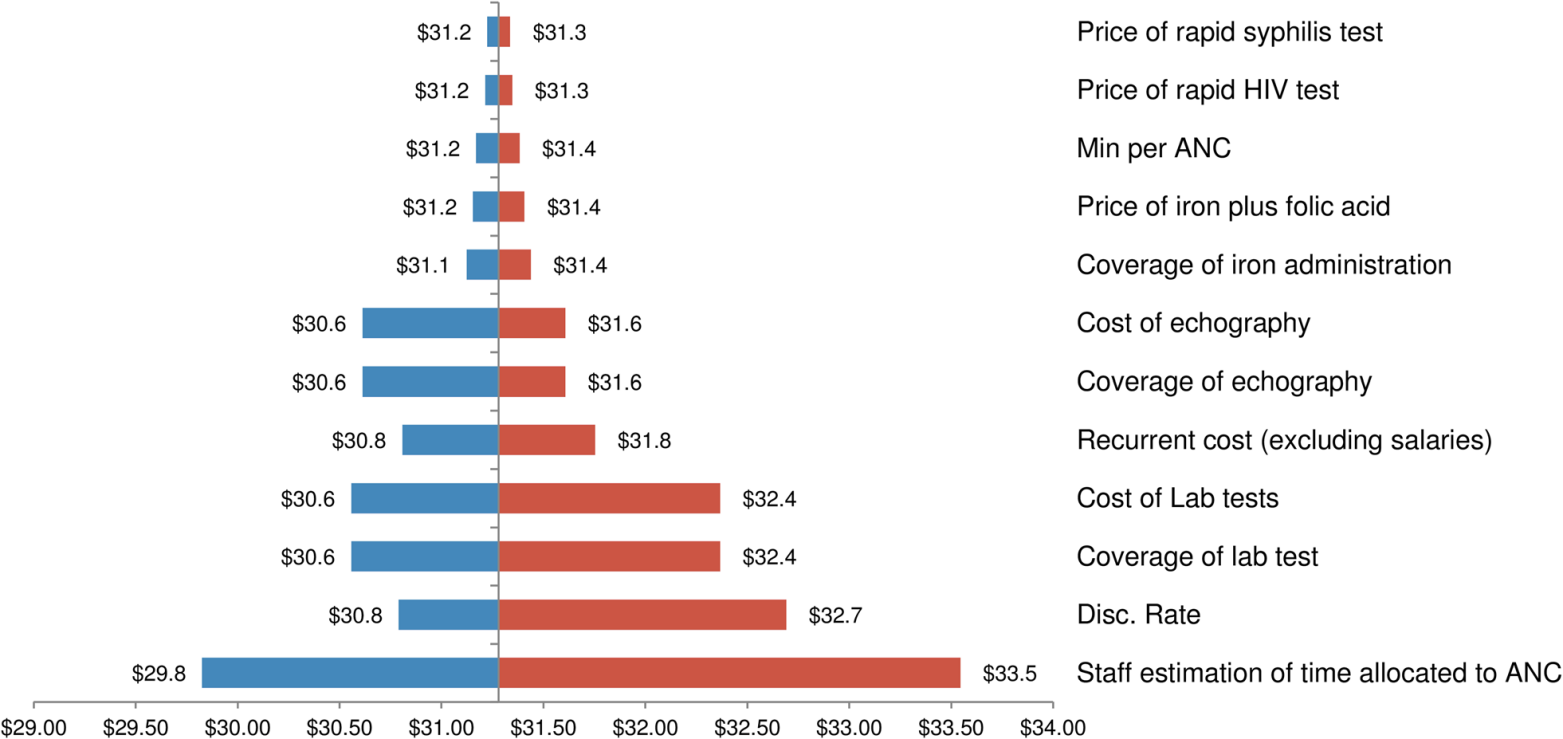
Tornado diagram of the univariate analysis of the full ANC cost per visit

Univariate sensitivity analysis showed that results were most sensitive to changes in personnel costs. Increasing staff time allocations by 20 % increased the unit ANC visit cost by $4.7 and decreasing this estimation by 20 % decreased it by $7.2 (see tornado diagram in Fig. 2).

**Fig. 3:**
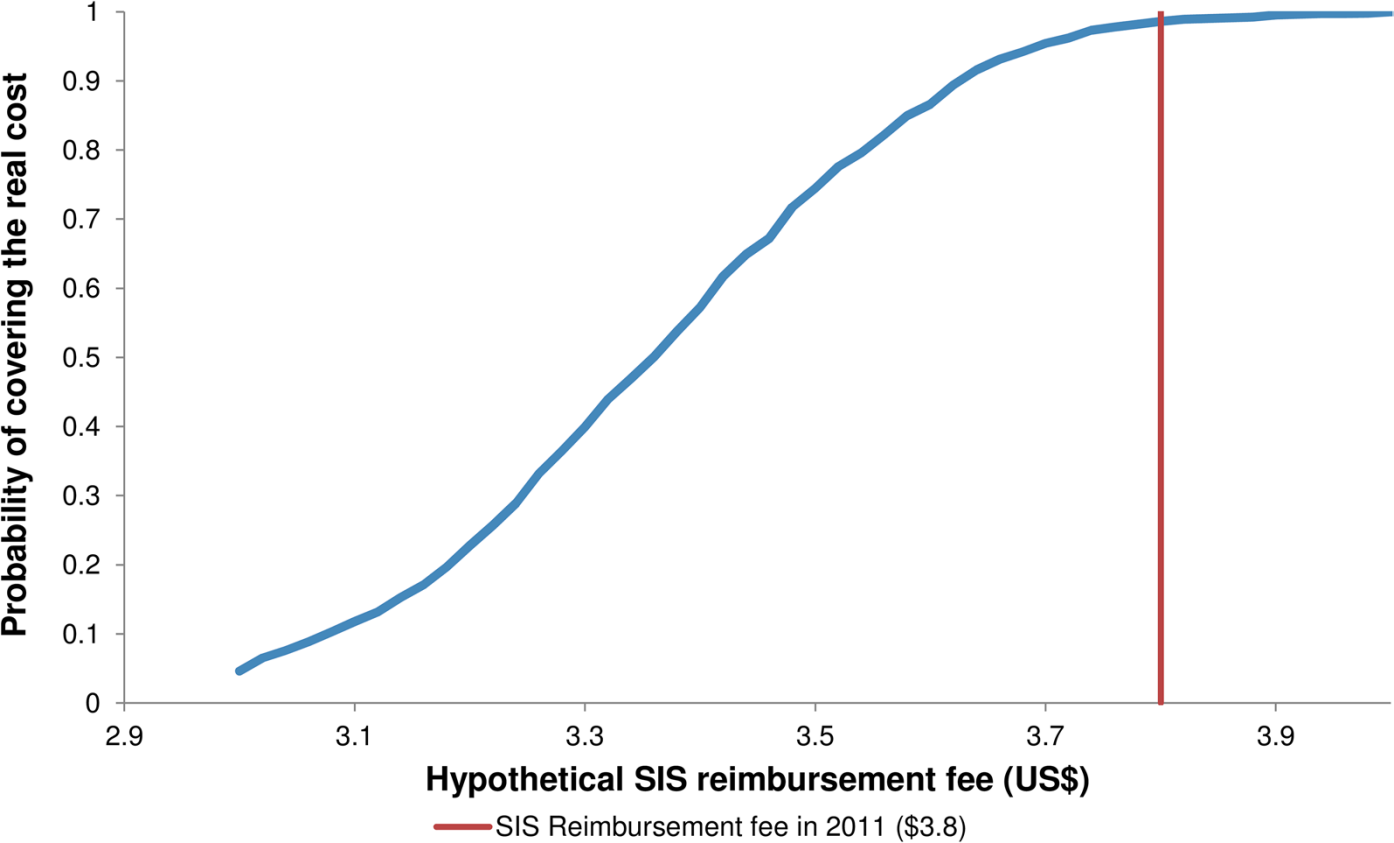
Probability of SIS reimbursement fee covers the real cost of the services. The figure presents different hypothetical reimbursement fees (X axis) and their probability of covering the real cost of the services using the results of the multivariate sensitivity analysis

We estimated the probability that the true cost of an ANC visit would be higher than the SIS reimbursement fees using the multivariate sensitivity analysis and found it to be 1.4 %. Thus, the reimbursement fee will cover the cost that it is supposed to cover in most of the cases. Figure 3 represents this relationship for different hypothetical reimbursement fees (X axis) using the results of the multivariate sensitivity analysis.

## Discussion

Our study of ANC provision in 8 health centres in Ventanilla District (Peru) found an average cost of $31.3 (95 %CI $29.7–$33.5) per ANC visit. These results are consistent with the findings of similar studies conducted in the region. Borghi et al. found an average cost for ANC visit of $31.1 in Rosario publicly maternity services in Argentina. In their study, personnel costs represented the highest proportion of total cost 91 % [20]. Cahuana et al. estimated the cost of different maternal and child interventions in Morelos state in Mexico. Here, ANC cost ranged between $38 and $55 depending on the cost estimation methodology used [21].

Comparing the different facilities in our study reveals that, although Ventanilla Baja had the lowest total costs of provision of ANC, it had the highest average cost per visit. In contrast, Mi Peru had one of the highest total costs of provision but it had the lowest average cost per visit, suggesting economies of scale. The range of unit visit cost seen amongst the different health centres can be explained based on the major cost drivers. As discussed, recurrent cost and personnel cost among them account for more than 70 % of the total cost of ANC provision. If we analyse the productivity of the midwives in terms of ANC visits per year (Table 6), we see that the lowest ratio is in Ventanilla Baja. In contrast, the highest ratio of visits per midwife per year is in Mi Peru, yielding the lowest cost per ANC visit. This relationship is also seen in Villa de los Reyes, Defensores, Santa Rosa and 3 de Febrero.

However, there were two health centres where midwives conducted high numbers of ANC visits but the unit ANC visit costs were also high (Peru Korea and Marquez). In Peru Korea, this may be due to the low number of ANC visits provided to each woman while, in Marquez it may be due to the high cost of the first visit. In addition, both these facilities have higher complexity (level I-4 according to the Peruvian Ministry of health) and this may mean that their operational costs are higher.

Thus, if the number of visits per woman is low (Peru Korea) or the cost of the first visit is significantly higher than the rest (Marquez or Ventanilla Baja) the unit cost of ANC provision rises.

We also aimed to compare the fee reimbursed by the SIS per ANC visit ($3.8) with the actual cost of the services that are supposed to be covered by this fee. We estimated that the actual cost of providing those services were on average $3.4 ($3.0–$3.9) per ANC visit. Our multivariate analysis allowed us to investigate how sensitive these results were to uncertainties in our input parameters and we found that in 98.6 % of cases, the fee reimbursed would cover the actual cost incurred by the health facility. This is not consistent with other published estimates where differences between SIS and the actual cost of other services provided varied between 7 % less and 32 % more than the SIS fee [6, 15, 22].

Our results suggest that the current system of ANC provision in Ventanilla District may be sustainable in the short run. However, a number of factors may affect the overall sustainability. Firstly, we found that the SIS fee did not cover the cost in three out of eight health facilities. This suggests a potential problem, although, due to our relatively small sample size, we cannot apply these findings to the country.

Secondly, SIS funds only account for slightly more than 10 % of the total cost of providing ANC services and their potential impact on the overall sustainability is limited.

Thirdly, current evidence suggests that since the launch of SIS in 2002, a significant number of people have gained access to services in public health facilities (ANC service and other) [23, 24]. In addition, a number of other interventions are being implemented in the region to further increase uptake of different preventive services. The budgetary implications of this increased demand are not well understood and they will need to be addressed in the near future.

Another issue of note is that SIS covers only ANC visits but no other clinical event during pregnancy, such as, a respiratory infection or a urinary infection. Treatment for these events is covered by central funds from the DIRESA and individual health facility funds.

Finally, the share allocated by the MoH to each region is usually based on historical budgets and it is difficult to see dramatic changes in short periods of time [15]. While SIS funds are directly dependant on the number of services provided, MoH funds are less flexible to adapt to the increased demand (i.e. recruitment of new staff usually takes months in the public system). Therefore, there is a risk of progressively impoverishing the public health network as demand increases. Although neither this nor the volume of resources involved have been properly documented by the regional offices, SIS funds have been used as a stopgap to pay other recurrent costs such as, personnel cost in order to meet the demand of curative and preventive services [6].

The system would benefit from stronger coordination among the different funding sources and from a continuous monitoring and evaluation of the resources needed in individual health facilities. For instance, the MoH should be willing and able to transfer extra salary funds to those health centres that experience demand beyond their capacity. Similarly, there should be regular assessments of the adequacy of capital resources used and increased funding provision for major refurbishments, extensions or completely new health centres buildings.

There are a number of limitations to our study. First, the cost estimates calculated are based on actual resources spent and recorded in the accounting records of the DIRESA. However, some facilities were under-resourced. Therefore, a low cost per visit could reflect insufficient resources rather than efficiency.

Second, the DIRESA accounting system is weak and some of the costs are not properly captured. Therefore, some cost information has been estimated by senior officials at DIRESA. Nevertheless, according to the information provided by the Regional Health Office, these represent the opportunity costs of the use of those resources.

Third, we have used a non-randomised purposive sampling to identify the facilities included in the study that could limit the external validity of our results. However, we included in our sample facilities of different degree of complexity and from all four sub-networks in Ventanilla District.

Lastly, we restricted our analysis to health provider costs, potentially underestimating the true cost of ANC visits.

## Conclusions

The cost of providing ANC services is consistent with other estimates in the region with the biggest proportion comprised of personnel cost. Our analysis suggests that the fee reimbursed by the Seguro Integral de Salud for each ANC visit is enough to cover the cost incurred by the health facility. However, there are significant threats to medium and longer term sustainability of this system as fee transfers represent a small fraction of the total cost of providing ANC. Increasing ANC coverage would require greater flexibility in other funding sources to the DIRESA in order to meet an increased demand.

## Additional file

**Additional file 1.** General characteristics of Ventanilla District health centres, source of information of cost categories collected, cost allocation criteria and data collection tools.
