# Supplementary material for: Matching comprehensive health insurance reimbursements to their real costs: the case of antenatal care visits in a region of Peru
Source: Cost Eff Resour Alloc. 2015 Sep 19;13:16. doi: 10.1186/s12962-015-0042-z (PMC4575779; doi:10.1186/s12962-015-0042-z)
Supplement: Supplementary file 1 — Additional file 1. General characteristics of Ventanilla District health centres, source of information of cost categories collected, cost allocation criteria and data collection tools. [file 12962_2015_42_MOESM1_ESM.docx]

| **Table S-1: General Characteristics of the health centres** | | | | | | |
| --- | --- | --- | --- | --- | --- | --- |
| Health Centre | Complexity^a^ | | Reference population | Total ANC visits 2011 | Opening hours | Included in the study |
| Subarea 3 DE FEBRERO | | | | | | |
| Peru- Korea Pachacutec | I - 4 | 13,929 | | 3,084 | 24hr | X |
| 3 de Febrero | I - 3 | 31,156 | | 2,543 | 12hr | X |
| Bahía Blanca | I - 2 | 14,062 | | 1,273 | 12 hr |  |
| Ciudad Pachacútec | I - 2 | 11,434 | | 2,451 | 12 hr |  |
| Subarea ANGAMOS | | | | | | |
| Angamos | I - 2 | 28,706 | | 2,478 | 12hr |  |
| Hijos del Almirante Grau | I - 2 | 26,467 | | 2,382 | 12hr |  |
| Defensores de la Patria | I - 2 | 15,354 | | 1,344 | 6hr | X |
| Ventanilla Alta | I - 2 | 20,991 | | 1,800 | 12hr |  |
| Mi Perú | I - 2 | 51,243 | | 4,194 | 12hr | X |
| Subarea LUIS FELIPE | | | | | | |
| Luis Felipe de las Casas | I - 2 | 11,854 | | 1,668 | 12hr |  |
| Villa de los Reyes | I - 3 | 18,910 | | 2,169 | 12hr | X |
| Sta. Rosa de Pachacútec | I - 2 | 15,557 | | 2,270 | 12hr | X |
| Subarea MARQUEZ | | | | | | |
| Márquez | I - 4 | 8,777 | | 2,136 | 24hr | X |
| Ventanilla Baja | I - 2 | 5,312 | | 480 | 12hr | X |
| Ventanilla Este | I - 2 | 7,854 | | 810 | 12hr |  |
| * Complexity refers to the range of services provided being I-2 the most simple. See Technical Note from MoH of Peru number 1142/2004 for more information. | | | | | | |

| **Table S-2: Allocation criteria in the step-down methodology** | | | |
| --- | --- | --- | --- |
| Cost | Allocated to | Allocation criterion | Source of information |
| **Step 1: Allocation of joint cost to all services** | | | |
| Buildings and maintenance | All services | According to space used | Building plans  Interview with staff |
| Equipment and furniture | All services | According to use | Inventory |
| Vehicles and Motorbikes | All services | According to use | Interview with driver |
| Salaries | All services | Where staff work | Interview to staff |
| Medicine and Pharmaceutical | Not allocated. Cost captured in bottom up methodology | | |
| Office Consumables* | All services | 25% overhead services  15% support services  60% final services | Interview with staff  Health statistics |
| Cleaning materials | All services | According to space | Building plans |
| Utilities & Telephone | Administration | All |  |
| **Step 2: Allocation of cost of overhead services to support and final services** | | | |
| Administration and accounting | All services | According to cost of each service in the first step | First step |
| Logistics and maintenance, cleaning and security | All services | According to space and use | Building plans  Interview with staff |
| Health information | All services | 5% to administration  5% to accounting  90% to support and final services according to the proportion of patients attended in each service | Interview with staff  Health statistics |
| **Step 3: Allocation of support services to final services** | | | |
| CHI office, admission and dispensary services | All final services | Proportion of patients attended in each service | Health statistics |
| Laboratory services | Outpatient services (including emergency room) and ANC services | 80% to outpatient services  20% to ANC services | Interview with staff. |
| Echography | Not allocated. Captured in the bottom up costing. | | |
| **Step 4: Allocation of final services to ANC provision**** | | | |
| ANC service, triage, immunization, heath promotion, dental care, nutrition and psychiatric services. | ANC care | Proportion of working week used to provide the ANC interventions in each service | Interview with staff |
| Other final services | Not allocated to ANC care | | |
| * According to proportion of patients attended in each support or final service  ** Final services were allocated to ANC because some activities included in the ANC protocol are provided by services different than the ANC service (e.g. vaccination) | | | |

| **Table S-3: Cost categories collected at the health centres and sources of information** | |
| --- | --- |
|  | **Source and type of information** |
| **Capital** |  |
| Buildings | Source: DIRESA. Details on the size and distribution of rooms were identified with the building plan. Due to significant differences in the price per square meter of construction for the different health centres, the median annualised cost per square meter constructed was used for all of them ($805.1/m^2^). To calculate the building cost of the ANC office, the total number of square meters of the ANC office was multiplied by the median cost per square meter. |
| Equipment | Inventory undertaken in 2010. Replacement cost provided by the DIRESA |
| Vehicles and Motorbikes | Inventory undertaken in 2010. Replacement cost provided by the DIRESA |
| Furniture | Inventory undertaken in 2010. Replacement cost provided by the DIRESA |
| **Recurrent** |  |
| Salaries | Source: Human Resources Department at DIRESA. Salary plus fringe benefit for each person working in the health centres |
| Medicine and Pharmaceutical | Price of tests and drugs used for ANC interventions provided by the DIRESA |
| Building and Maintenance | Estimated by the DIRESA for each health centre |
| Office Consumables | Estimated by the DIRESA for each health centre |
| Cleaning materials | Estimated by the DIRESA for each health centre |
| Utilities & Telephone | Estimated by the DIRESA for each health centre |

| **Table S-4: % of the cost of the service allocated to ANC. Based on interviews with personnel** | | | | | | | | |
| --- | --- | --- | --- | --- | --- | --- | --- | --- |
|  | 3 DE FEBRERO | DEFENSORES | MARQUEZ | MI PERU | PERU COREA | SANTA ROSA | VENTANILLA BAJA | 3 DE FEBRERO |
| Immunization | 15 | 10 | 8 | 10 | 30 | 10 | 5 | 10 |
| Health promotion | 10 | 15 | 15 | 15 | 15 | 15 | 15 | 15 |
| Nutrition | 15 | 20 | 25 | 25 | 30 | 20 | 20 | 15 |
| Triage | 50 | 20 | 15 | 20 | 5 | 20 | 15 | 20 |
| Outreach services | 0 | 0 | 0 | 0 | 0 | 0 | 0 | 20 |
| Dental care | 15 | 50 | 8 | 25 | 30 | 25 | 15 | 20 |
| Psychiatric services | 25 | 25 | 30 | 35 | 40 | 40 | 20 | 20 |
| Estimated based of the amount of time that the staff working on each specific service spent on average on ANC activities. | | | | | | | | |

**Table S-5: Data collection tools**

|  | TOTAL HEALTH CENTRE |
| --- | --- |
| Salaries |  |
| Medicine and Pharmaceutical |  |
| Non Established Posts (casual labour) |  |
| Travel and Transport |  |
| Building and Maintenance |  |
| Non Drugs Consumables |  |
| Printing & Stationary |  |
| Office Consumables |  |
| Cleaning materials |  |
| Telephone |  |
| Electricity/water |  |
| Training & Conferences |  |
| **TOTAL RECURRENT** |  |
|  |  |
| Buildings |  |
| Equipment |  |
| Vehicles and Motorbikes |  |
| Furniture |  |
| **TOTAL CAPITAL** |  |
|  |  |
| **TOTAL GENERAL** |  |
|  |  |
| Health facility total square meters |  |
| ANC office square meters |  |
| Price per square meter |  |
| Price of Iron |  |
| Price of a rapid HIV test |  |
| Price of RPR |  |
| SIS reimbursement fee for standard laboratory tests for pregnant women |  |
| SIS reimbursement fee for standard ultrasound for pregnant women |  |
|  |  |
| Full inventory of the health facility |  |
| Salary + fringe benefits of all the staff involved in ANC |  |
|  |  |
| Total number of pregnant women attended |  |
| Total number of ANC visits provided |  |
| Total number of HIV/RPR test performed |  |
| Total number of pregnant women that received iron |  |
